# Effect of Cement Type on the Mechanical Behavior and Permeability of Concrete Subjected to High Temperatures

**DOI:** 10.3390/ma12183021

**Published:** 2019-09-18

**Authors:** Izabela Hager, Tomasz Tracz, Marta Choińska, Katarzyna Mróz

**Affiliations:** 1 Cracow University of Technology, Faculty of Civil Engineering, Chair of Building Materials Engineering, 24 Warszawska St., 31-155 Cracow, Poland; 2 Nantes University—IUT Institut Universitaire de Technologie, Research Institute in Civil and Mechanical Engineering GeM—UMR CNRS Unité Mixte de Recherche du Centre National de la Recherche Scientifique 6183, 44600 Saint-Nazaire, France

**Keywords:** high temperature, damage, permeability, CEMI and CEMIII, mechanical properties

## Abstract

The paper presents experimental investigations concerning the influence of the cement type (CEMI 42.5 R Portland cement and CEMIII/A 42.5 N slag cement—with 53% granulated blast furnace slag) on the mechanical and transport properties of heated concretes. The evolution of properties due to high temperature exposure occurring during a fire was investigated. High temperature exposure produces changes in the transport and mechanical properties of concrete, but the effect of cement type has not been widely studied in the literature. In this paper, concretes were made with two cement types: CEMI and CEMIII, using basalt (B) and riverbed aggregates (RB). The compressive and tensile strength, as well as the static modulus of elasticity and Cembureau permeability, were tested after high temperature exposure to 200, 400, 600, 800, and 1000 °C. The evaluation of damage to the concrete and crack development due to high temperature effects was performed on the basis of the change in the static modulus of elasticity. The test results clearly demonstrated that permeability increases with damage, and it follows an exponential type formula for both types of cement.

## 1. Introduction

Cements with granulated blast furnace slag are widely employed due to their lower carbon footprint, as a strategy for sustainable development in the field of construction. The use of ground granulated blast furnace slag (GGBFS), which presents an amorphous structure and shows pozzolan characteristics, in concrete as an additive has a positive effect on the properties of fresh and hardening concrete [1,2]. The use of GGBFS provides the important advantage of helping to avoid thermal cracks in concrete due to the low hydration process [2]. In fact, as previous results have shown, the hydration of GGBFS is slower than that of ordinary CEMI cement. Concrete with ground granulated blast furnace slag has a later setting time and a lower stiffness [3].

When equal amounts of cement and water binder (w/b) are used, concretes with slag content have a lower compressive strength at early ages and higher compressive strength at late ages than Portland cement [2]. Furthermore, with a specific compressive strength, slag concrete has a better mechanical performance in terms of tension than concrete made with Portland cement [2]. However, the study by Shumuye et al. [4] showed that the compressive strength of the concrete decreased as the slag content increased.

Environmental conditions and the temperature exposure during curing has a strong effect on concrete mechanical properties [3,5]. When the material is subjected to heating at higher temperatures up to 1000 °C, like during a fire, thermal damage occurs due to dehydration of the cement paste and the thermal mismatch of strains between the shrinking cement paste and expanding aggregates, which induces cracking [6,7]. Moreover, during the phase of cooling down to the ambient temperature, stresses induced by inversed thermal gradients result in the development of cracks within the cement paste that will affect permeability, and also may compromise the durability of the material after a fire [8,9]. The changes of concrete’s mechanical properties at high temperatureshave been widely investigated [6,7,8,9,10,11,12], helping us to better understand the behavior of concrete structuresin a fire situation and to determine parameters influencing its behavior. The evolution of concrete mechanical properties in fire depends on the concrete composition: presence of mineral additions [4,13], w/c water cement ratio [14,15], the nature and type of aggregates [10,11,12]. Moreover, the concrete heating conditions: heating rate and maximum temperature of exposure play a major role in concrete strength evolution, as well as the testing procedure: hot tested concrete or tested after temperature exposure and cooling down to the ambient temperature [7,15]. Nevertheless, for material mechanical properties testing, a slow heating rate is recommended in order to ensure limitation of the thermal gradient inside the specimen. In the literature investigations the heating rates of 0.1–10 °C/min are employed. Nevertheless, the heating rates recommended by RILEM International Union of Laboratories and Experts in Construction Materials, Systems and Structures [16] depend on the specimen diameter and are from 0.5 to 2.0 °C/min for accidental conditions (fires). 

The existing knowledge regarding the behavior of high performance concrete in a fire was recently reviewed by the RILEM Technical Committee HPB-227 [8], however, there are still no clear reports as to whether the properties of concretes subjected to high temperatures change in a similar or a very different way, depending on the cement type used.

According to Shumuye et al. [4], the addition of GGBFS seems to improve the resistance of concrete to fire conditions. It was highlighted that, when the exposure to fire temperature increased from 200 to 400 °C, the compressive strength increased for concrete with slag (70% ordinary Portland cement OPC and 30% slag cement, as well as 50/50 proportions). For the group of concretes with 30% OPC and 70% slag cement, the opposite behavior was observed. The concrete mix containing GGBFS usually has a lower thermal expansion coefficient than Portland cement. The 15% and 30% replacement of CEMI by GGBFS gives coefficients of thermal expansion of 22.7 × 10^−6^/°C and 17.2 × 10^−6^/°C, respectively, which is 99.2% and 75.5% of the value obtained for Portland cement paste [4]. However, a recent study by Asamoto et al. [17] highlighted that the reduction in the elastic modulus and increase in permeability of the concrete with GGBFS subjected to 65°Cwere larger than those of concrete without slag. Indeed, astonishingly, this can be attributed to a larger thermal expansion coefficient and larger cement paste shrinkage with the slag, leading to the formation of microcracks around the aggregate.

Moreover, it can be concluded that the addition of aluminosilicate minerals like fly ash, ground granulated blast furnace slag (GGBFS), and silica fume (SF) can affect concrete behavior at high temperatures in a way that may produce spalling of heated concrete in material that is denser, and thus less permeable [18,19,20]. Lower permeability leads to moisture clog occurrence and increase of vapor pore pressures inside the heated concrete [20]. The moisture clog effect was explained and linked with the permeability decrease observed in temperature from 100 to 200 °C but this effect is observed when the permeability is tested at hot stage and not after cooling down when the residual values of permeability are determined, like in present study. An important finding on gas pore pressure development were provided by works of Kalifa et al. [19,20] and linked with the permeability.

Cases of fires that took place in engineering facilities (Gotthard tunnel, Chunnel tunnel, or Mont Blanc tunnel, for example) have caused numerous fatalities, but also significant financial losses. During these fires an important loss of concrete in tunnel linings was observed. The load-bearing capacity of the structural elements was reduced due to the explosive spalling. The spalling may take different forms, from small concrete pieces chipping, known as the popcorn effect, to explosive behavior when larger pieces of concrete are separated from the concrete element with great energy [21,22,23,24]. In all cases, concrete fire spalling leads to the exposure of steel reinforcement, which is sensitive to high temperatures [24,25]. So far, it has been confirmed that the type and composition of concrete, including the aggregate type, water cement ratio, pozzolanic mineral material, and moisture content of concrete, affect its behavior in fire conditions [8,13,19]. Research aimed at understanding the causes of the spalling phenomenon, as well as determination of material parameters affecting its intensity, has been carried out by experiments [21,22,24] and numerical analysis [21,25,26]. Thus, concrete spalling is one of the most interesting and complex phenomena occurring in concrete exposed to fire conditions. The RILEM Technical Committee 256-SPF: Spalling of concrete due to fire: Testing and modelling has been established, and is mainly dedicated to studying this specific behavior. 

During heating, the permeability usually progressively increases [27,28,29], exceptwhenthe permeability of concrete may decrease [30] due to the moisture clog effect. In this situation the water vapor pressure increases in the material’s pore network, which may lead to spalling behavior. It is believed that the interaction of high temperature, an increase in water vapor pressure in the material pores, and the internal stress state is responsible for the occurrence of concrete spalling [19,20,21,22,23,24,25,26]. It seems that the key parameter governing the occurrence of spalling is its permeability. In denser and less permeable concretes the risk of spalling is higher. Researchers have shown that in fire conditions, concretes that are modified with the addition of mineral additives like silica fume and calcareous filler are prone to spalling behavior. As the spalling behavior of concrete is mainly governed by its permeability, researchers have been testing the influence of GGBFS addition on concrete permeability. Recently, Karahan [27] showed an increase of concrete transport properties after exposure to temperatures of 400 °C, accompanied by compressive strength reduction. Moreover, the conclusion of the authors indicated an optimum GGBFS/cement blend from the point of view of material behavior in a fire of 50–70% slag content as the cement replacement.

Hence, the results available do not reflect all the relevant aspects of this topic, and additional investigation is required. The literature results cannot be compared to each other due to the fact that the mixes differ. A research programme was therefore proposed which would allow for a clear comparison of the influence of cement type on the mechanical and physical properties of concrete at high temperatures. For this we performed various tests on identical concrete mixes, for which the only changing factor was the cement. Therefore, the main goal of this work is to present the comparison of the changes in mechanical and physical properties of concretes made with two different cement types; CEMI and CEMIII. For all four concretes, the composition of cement paste, as well as the volume of cement paste and mortar, remained the same. Thus, the study reflected solely the cement type effect of Portland cement versus slag cement on the mechanical performances and permeability of concretes made with two types of aggregates: crushed basalt (B) and riverbed gravel (RB). For all the concretes tested, the amount of all components (cement paste and mortar volume) and aggregate type and nature, as well as the particle size distribution, was identical, apart from the type of cement. 

This research investigates the mechanical performances and permeability of concretes made with different cements, to compare their reference mass transport capacities, strength, and stiffness after high temperature exposure. The reference values of permeability enable one to assess their potential for spalling in fire conditions, as denser and less permeable materials are prone to this behavior. Furthermore, the evolution of permeability with heating temperature was investigated, as well as the compressive strength and splitting tensile strength. Moreover, the stress strain curves were determined, and the modulus of elasticity was determined. All residual mechanical performances (f_cT_, f_tT_, E_T_) were evaluated after heating to temperature T (°C), which corresponds to the post-fire performance of concrete in situations where the assessment of material properties is required. In this specific situation, the residual permeability of concrete is also an issue because it governs all aspects of durability, and there may be a need for assessment when a decision must be made on the further use of concrete elements after a fire.

## 2. Materials, Specimen Preparation, Curing, and Heating

The concretes investigated in this research were manufactured with the following components: Portland cement CEMI 42.5R and CEMIII/A 42.5 N containing 53% GGBFS, quartz sand 0/2mm, and one of two types of coarse aggregate: (B)basalt or (RB) riverbed gravel.

Cements from Lafarge (Małogoszcz, Poland) were used for both the CEMI 42.5 R Portland cement and CEMIII/A 42.5 N slag cement. The chemical characteristics of these cements are provided in Table 1, the physical characteristics in Table 2, and the mechanical characteristics in Table 3.

Two types of aggregates were used in this research programme: gravel from Dunajec River (Dwudniaki, Poland) and crushed basalt.

In Table 4, the concrete mixes are presented. The cement paste volume was 300 dm^3^/m^3^ and the mortar volume was 550 dm^3^/m^3^. The concretes are denominated as B CEMI, B CEMIII, RB CEMI, and RB CEMIII. Plasticizer (BASF BV 18 (Myślenice, Poland) and superplasticizer (BASF Glenium SKY 591 (Myślenice, Poland) were used and the water-cement ratio (w/c) of the concretes was equal to 0.3.

All concrete cubic and cylindrical specimens were cast in plastic molds and stored for 24 h. After preliminary 24 h curing, the molds were covered with plastic lids for 7 days to prevent water evaporation. Samples were stored in laboratory conditions at T = 20 ± 5 °C and relative humidity HR = 50% ± 5%. Cylindrical specimens dedicated to permeability measurements were cut into discs with a diameter of 150 mm and thickness of 50 mm at the age of 28 days. At 90 days, all specimens for mechanical performance testing and permeability were heated in an electric furnace to T = 200, 400, 600, 800, and 1000 °C. As recommended by RILEM [16], a heating rate of 0.5 °C/min was applied. A slow heating rate is applied for concrete mechanical behavior testing at high temperatures in order to ensure limitation of the thermal gradient inside the specimen. When the target temperature was reached it was maintained for three consecutive hours in order to obtain a homogenous temperature in the whole cross section of the specimen. Afterwards, all specimens were cooled down inside of the furnace chamber.

## 3. Testing Procedures

### 3.1. Concrete Permeability

**Figure 1:**
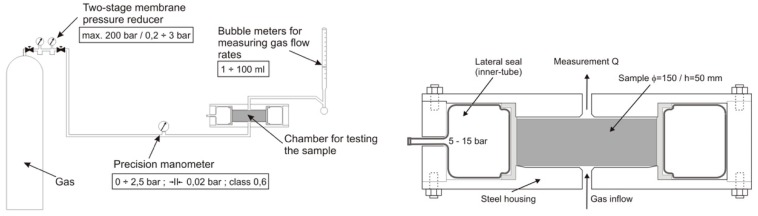
Permeability Cembureau testing set-up of the sample chamber.

The permeability test used nitrogen as a gas media and the Cembureau method was applied [31]. The testing set-up used is presented in detail in Figure 1.

In Equation (1), the permeability (k) was determined:

(1) $$k=\frac{2{\mathrm{QP}}_{a}\eta L}{A\left(P^{2}-P_{a}^{2}\right)}\left(m^{2}\right),$$

where:

Q: the measured gas flow intensity Q = V/t (m^3^/s);
V: gas volume (m^3^)
t: time (s)
P_a_: atmospheric pressure (1 bar = 10^5^ Pa);
P: absolute pressure (Pa);
A: cross section area of the specimen (m^2^);
η: nitrogen viscosity; η = 17.15 (Pa⋅s);
L: thickness of the specimen (m).

The initial reference permeability of the concrete in the samples at 90 days old was determined on the specimens that were not pre-dried, in order to represent the non-dried condition in the real structure. Subsequently, the samples were heated to a temperature ranging from 200 to 1000 °C, and after cooling the permeability was measured. Each measurement value represented the mean value from three samples.

### 3.2. Mechanical Tests

The cubic specimens with side a = 150 mm were used for compressive strength determination, with a diameter (d) of 100 mm and height (h) of 200 mm for the cylindrical samples for the splitting tensile strength tests. Three samples were used to test unheated concrete and two were used to test heated concrete. The modulus of elasticity was determined from the stress–strain (σ-ε) using one cylindrical sample (d = 100 mm; h = 200 mm). All E values were expressed in GPa and calculated from σ-ε curves as the stress to strain and strain ratio in the range of 10% to 40% of the ultimate stresses. For all properties six temperature levels were studied: T = 20, 200, 400, 600, 800, and 1000 °C. The compressive strength test procedures applied were presented in EN 12390-3 [32], and the splitting Brazilian tests were done according to EN 12390-6 [33]. 

## 4. Test Results and Discussion

### 4.1. Initial Properties

For B CEMI, B CEMIII, RB CEMI, and RB CEMIII concretes, the initial physical properties of bulk density ρ_o20°C_ and permeability k, and the mechanical properties of compressive strength f_c20°C_ tensile strength f_t20°C_ and modulus of elasticity E_20°C_ were determined after 90 days. The initial measurements, obtained for non-heated concrete properties, are presented in Table 5 and marked with the symbol 20 °C.

### 4.2. Evolution of Bulk Density with Temperature 

**Figure 2:**
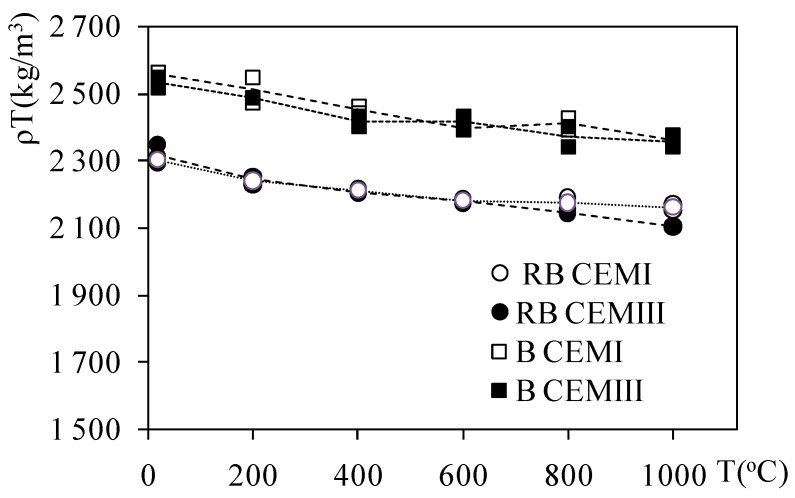
Bulk density of riverbed aggregates (RB) and basalt (B) concretes made with CEMI and CEMIII concretes; mean value of three samples.

The progressive increase of the temperature resulted in free water evaporation and progressive dehydration of the material. The C-S-H, as well as portlandite and calcium carbonate decomposition, were progressive in higher temperatures. As a result, weight loss was observed and the progressive density changes were recorded. The bulk density of B CEMI, B CEMIII, RB CEMI, and RB CEMIII concretes decreased as a function of the temperature. The mean values of bulk density are presented in Figure 2.

In Figure 2 the bulk densities of the test concretes are presented. The values are mainly related to the type of aggregate: basalt or riverbed. The density of basalt CEMI concrete was 2558.8 kg/m^3^ and the B CEMIII 2533.2 kg/m^3^. The RB CEMI and RB CEMIII concrete were 2300.7 and 2315.6 kg/m^3^, respectively. Apart from the initial values of density observed in the non-heated pristine concrete, the changes of the density with the temperature were quite similar for both cement types.

### 4.3. Evolution of Compressive Strength and Splitting Tensile Strength with Temperature Exposure

**Figure 3:**
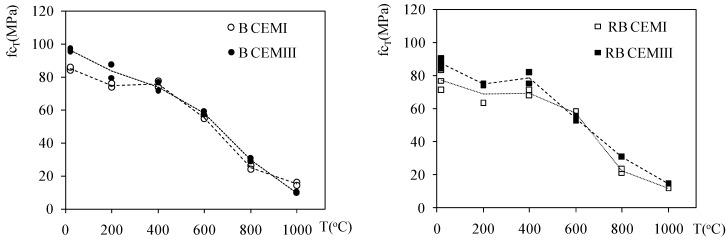
The compressive strength evolution CEMI and CEMIII concretes on basalt and riverbed aggregate.

Figure 3 depicts the average and individual values of compressive strength. From the figure it can be concluded that the compressive strength of unheated concrete was higher for both CEMIII concretes made with basalt and riverbed aggregates. This tendency is maintained at 200 °C. When the temperature is higher than 400 °C, there are few differences in strength between B CEMI and B CEMIII, as well as between RB CEMI and RB CEMIII concretes. They all presented almost the same strength of 60 MPa.

**Figure 4:**
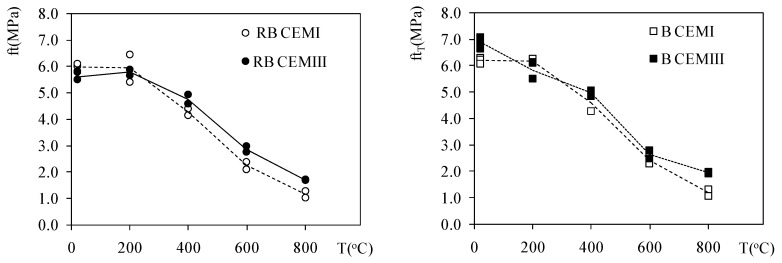
The changes in the splitting tensile strength of heated CEMI and CEMIII concretes on basalt and riverbed aggregate.

In Figure 4, the average and individual values of f_tT_ are presented. Heating resulted in a progressive reduction in strength, nevertheless, the differences between CEMI and CEMIII concretes over a whole range of temperatures may be considered insignificant, in the scope of measurement error, or the scatter of results for this mechanical property. 

As has already been shown in previous research, an important aspect in the high temperature behavior of concrete is the thermal stability of aggregates at high temperatures. This can be evaluated by thermo-gravimetric and differential thermal analysis, which indicate the physical or chemical transformation of aggregates. As has already been reported [10], basalt is thermally stable up to 1000 °C; above this temperature melting is observed at 1050 °C and expansion and gas release both occur.

### 4.4. Relationship between Stress and Strain, and the Modulus of Elasticity Evaluation

**Figure 5:**
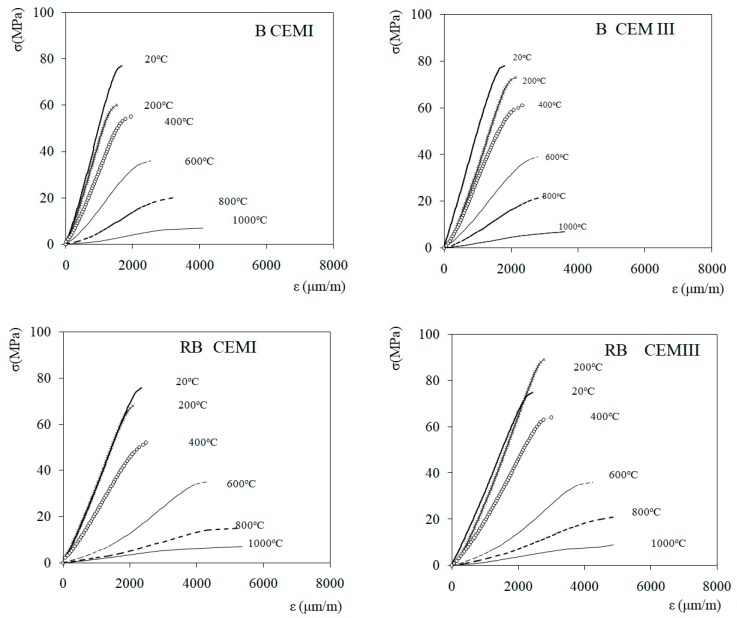
curves of heated concretes.

The stress–strain relationships for the tested concretes are presented in Figure 5. Along with the temperature increase, a change of concrete stiffness was observed, as represented by the slope of the stress–strain curve. For the specimens heated to 600 °C and above, the stress–strain curve presents nonlinear behavior in compression due to the presence of cracks, which are closing partially when a compressive load is applied during the test. The similar stress–strain behavior of concrete in compression was observed for hot tested and tested after cooling down [8,15], an important cracking of samples was observed, especially for concretes with siliceous aggregates, heated without loading. The cracking of unloaded concrete was confirmed by the thermal strain evolution observation during heating [6].

**Figure 6:**
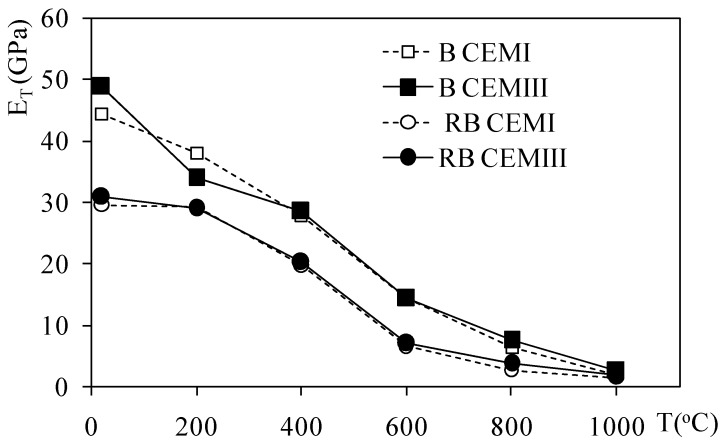
Modulus of elasticity change with the temperature of CEMI and CEMIII concretes.

The static modulus of elasticity values (E_T_) of heated B CEMI and B CEMIII, as well as RB CEMI and RB CEMIII, are shown in Figure 6. The pristine non-heated concretes’ modulus of elasticity (E_20°C_) were 44.4 and 48.9 GPa, respectively, for B CEMI and B CEMIII. For riverbed aggregate RB CEMI and RB CEMIII they were 30.6 and 29.7 GPa. These results show clearly that for concretes with the same volume of cement paste, the modulus of elasticity is related to the nature of the aggregate and is strongly related to concrete density. Higher values of E_T_ were observed for both CEMIII concretes with RB and B aggregates.

A quasi linear decrease in the E_T_ value over the whole range of heating temperatures was observed. The slope of E_T_ decrease is most pronounced in the range of temperatures between 400 and 1000 °C (Figure 5). This sharp decrease of stiffness was attributed to crack development due to a mismatch of the strains between the cement paste and aggregates that is observed in this range of temperatures, and an increase in thermal strains resulting from cracking [6,7].

From Figure 6 it can be concluded that the relative change of the modulus of elasticity is quasi identical for the concretes tested, and does not depend on cement type. The differences between the modulus of elasticity values of RB CEMI and RB CEMIII are not significant except for differences occurring at 20 °C.

### 4.5. Heated Concrete Permeability Evolution

**Figure 7:**
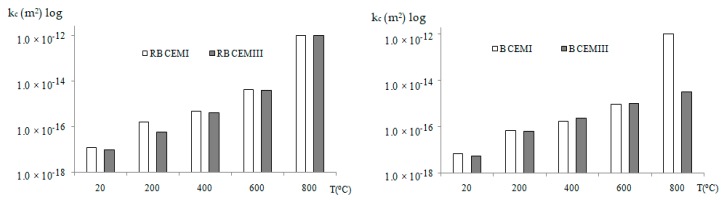
Effect of heating on the permeability of the test materials: RB CEMI and RB CEMIII, B CEMI and CEMIII. The reference permeability at 20 °C and permeability after heating to 200, 400, 600, and 800 °C.

For RB CEMI and RB CEMIII, the initial reference permeability, measured on non-heated concrete after exposure to 20 °C, reached values of 1.20 × 10^−17^ m^2^ and 1.00 × 10^−17^ m^2^, respectively. For B CEMI and B CEMIII this permeability was 0.70 × 10^−17^ m^2^ and 0.52 × 10^−17^ m^2^. With the increase of heating temperature residual permeability was increased. For the specimens heated to 1000 °C the permeability could not be measured due to crack development, and the gas flows could not be stabilized, so the permeability could not be measured with the Cembureau set-up. The results of the permeability measurements are presented in Figure 7. For B CEMIII and RB CEMIII concretes generally, lower values of permeability were observed. For the riverbed aggregate concrete RB CEMIII, permeability measured after exposure to high temperatures at 200, 400, 600, and 800 °C was systematically slightly lower than for RB CEMI. Basalt aggregate-based concretes provide lower permeability than riverbed ones. Nevertheless, these differences could not be considered significant. For all the concretes heated up to 1000 °C, the permeability could not be measured with the Cembureau method due to the significant damage to the concrete and crack development.

### 4.6. Permeability vs. High Temperature Damage Factor

Previous studies [34,35] have indicated that the degradation of concrete at high temperatures, arising from a coupled hygro-thermal, chemical (dehydration) and mechanical interaction, can be modelled by means of the isotropic damage theory of Mazars [36]. Following Gawin et al. [9], the total damage *D* may be described by a multiplicative format of mechanical and thermo-chemical damage components, as shown in Equation (2):

(2) $$D=1-\frac{E\left(T\right)}{E_{0}\left(T_{0}\right)}=1-\frac{E\left(T\right)}{E_{0}\left(T\right)}\frac{E_{0}\left(T\right)}{E_{0}\left(T_{0}\right)}=1-\left(1-d\right)\times \left(1-V\right),$$

where V corresponds to the thermo-chemical damage and *d* to the mechanical damage. The term (1 – d) corresponds to $\frac{E\left(T\right)}{E_{0}\left(T\right)}$, and (1–V) to $\frac{E_{0}\left(T\right)}{E_{0}\left(T_{0}\right)}$. In the above equation E_0_(T_0_) is the initial value of the static modulus of elasticity, E_0_(T) is the modulus for mechanically undamaged material expressed in a function of heating temperature, and E(T) represents the static modulus of elasticity of mechanically damaged heated concrete. 

**Figure 8:**
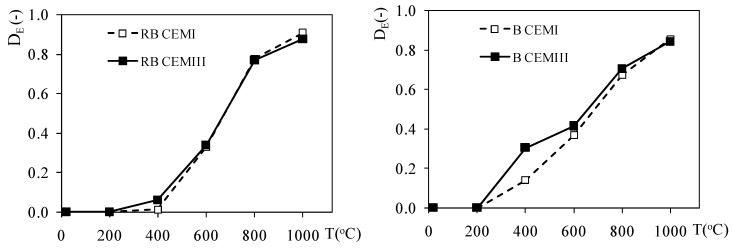
Damage factor (*D_E_*) as a function of temperature.

Following this approach, in Figure 8 the effect of temperature on the damage parameter for heated concretes is presented. The damage factor was calculated on the basis of the change in the modulus of elasticity with temperature (see Figure 6), leading to Equation (3), and this evaluates the deterioration of the stiffness of the heated concrete samples by comparing them with the parameters found in non-heated concrete:

*D_E_ = 1 − E_T_/E_20 °C_*, (3)

where E_20°C_ is the static modulus of elasticity tested at 20 °C and E_T_ is the value obtained for heated concrete.

The damage factor follows a comparable increasing change for all tested materials and almost reaches the value of 0.9, which means that 90% of the concrete has deteriorated. However, at 400 °C the damage value becomes much higher for the basalt aggregate concretes in comparison with the riverbed aggregate ones. Overall, the damage values for the CEMIII concretes appear to be slightly lower than for the CEMI concretes, especially for the basalt-based materials. 

These changes may be qualitatively compared to the change of total damage with temperature of a high performance concrete [9]. However, the damage values obtained and cited in this study are higher (damage of 0.8 at 600 °C). The reason for this difference may be due to the heating conditions, and notably the heating rate, which was four times higher in the study by Gawin et al. [9] than in our procedure, and which may provide stronger thermal gradients and therefore greater degradation.

**Figure 9:**
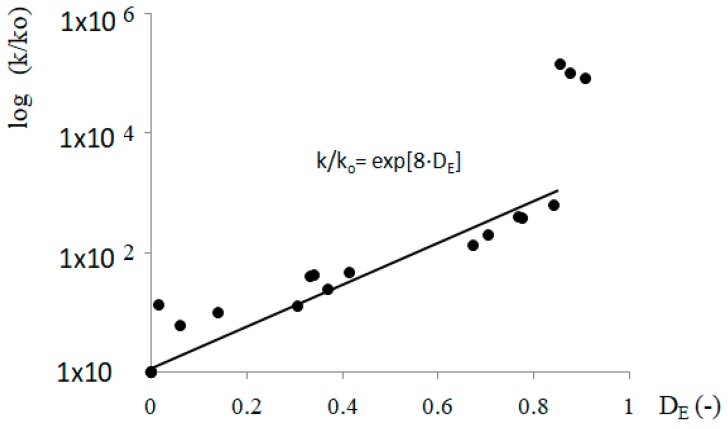
Juxtaposition of permeability (log scale) and damage to unheated concretes (20 °C) and concretes heated to 200, 400, 600, and 800 °C.

It has already been noted that the changes to the inner micro-structure and permeability of the concrete may be characterized using this mechanistic approach, using damage evaluation to describe the high temperature degradation and/or micro-cracking effects [9,26,37,38]. The results of such a correlation are presented in Figure 9 for all the test materials. One may observe that all the data follow a single master law, independent of cement type or aggregate type. The results follow an exponential relationship, except for the permeability values obtained at 800 °C (Equation (4)):

(4) $$\frac{k}{k_{0}}=\exp\left[C_{DE}\cdot D_{E}\right].$$

In Equation (4) k is the permeability of the heated material, k_0_ the initial reference permeability, D_E_ the damage factor, and C_DE_ is the material dependent parameter, here equal to 8, which confirms the value obtained for another high performance concreteat elevated temperatures, but based only on the CEMI cement [9]. The C_DE_ value being equal to 8was obtainedfrom the regression curve with the coefficient of determination R^2^ of 0.86 value. Therefore, the proposed regression curve is limited in the range of temperature from 20 to 600 °C.Three points that do not follow the trend in Figure 9 correspond to permeability values obtained at 800 °C. At this temperature, important cracking occurs following the already mentioned nonlinear mechanical behavior.

## 5. Conclusions 

This paper intended to present the study of the influence of the cement types CEMI and slag cement CEMIII, in which the GGBFS amount reaches 54%, on the mechanical and physical performances of heated concretes with riverbed (RB) and basalt (B) aggregates. Four concretes with the same volume of cement paste and mortar were investigated. The only parameter differentiating RB and B concretes was the cement type. Analysis of the experimental data obtained concerned the mechanical tests, stiffness, and the permeability test results of the four concretes subjected to high temperature exposure (up to 1000 °C). The following main conclusions were drawn:

1. Type of cement influences compressive strength and permeability of 90 day concrete. Concretes with CEMIII presented lower permeability and higher compressive strength for both basalt and riverbed aggregate concretes;
2. High temperature exposure strongly influences the mechanical and physical properties of concretes, and the damage to concrete increases with exposure temperature. A temperature increase leads to the reduction of strength and modulus of elasticity. The splitting tensile strength decrease is more pronounced than the compressive strength evolution.
3. Minor differences between the mechanical properties of heated CEMI and CEMIII concretes were observed. The bulk density values, as well as the mechanical properties f_cT_, f_tT_ and E_T_, were very close or the differences were within the range of measurement error or the scatter of results of the properties tested;
4. The nature of the aggregate has a dominant influence on the material physical density and mechanical properties of the tested concretes. The compressive and tensile strengths depend on the aggregate nature for temperature up to 400 °C; above this temperature level, similar values of strength are observed;
5. The decrease in the mechanical properties is the result of progressive cement paste damage due to dehydration and chemical changes in the cement paste. Moreover, crack development due to the thermal mishmash of aggregate and cement paste results in nonlinear behavior of heated concretes;
6. The course of changes of the relative value in the elastic modulus for all the concretes investigated was very similar, except for the temperature of 400 °C. The riverbed aggregate concretes RB CEMI and RB CEMIII hada lower damage parameter than that observed for basalt aggregate concretes (B CEMI and B CEMIII) at this temperature. For 200, 600, 800, and 1000 °C, the damage levels were similar;
7. Important changes of up to six orders of magnitude were observed in permeability values following heating. However, the differences between the concretes could not be considered as significant. Indeed, CEMIII concretes presented slightly lower values of permeability in comparison with the CEMI ones in whole range of temperatures. On the other hand, basalt aggregate-based concretes have slightly lower permeability than riverbed ones. Concretes with CEMI: riverbed 1.2 × 10^−17^ vs. basalt 0.7 × 10^−17^; concretes with CEMIII: riverbed 0.99 × 10^−17^ vs. basalt CEMIII 0.53 × 10^−17^. That difference was be explained by lower permeability of basalt aggregate itself. This relation was also observed for the temperatures of 200, 400, and 600 °C;
8. Analysis of the results allowed the formulation of the constitutive exponential law, presenting the relationship between the permeability of concrete and damage, which is valid up to 600 °C.
9. It can be considered that heating induces damage, which may be represented by changes in the initial modulus of elasticity, that depends to a small degree on the type of cement. In this range of damage, the effects of aggregate type are also non-significant.

## Figures and Tables

**Table 1:** The chemical characteristics of CEM I and CEM III cements(oxide analysis, % by mass).

Component	CEMI 42.5 R	CEMIII/A 42.5 N
SiO_2_	18.6	30.0
Al_2_O_3_	5.3	6.2
Fe_2_O_3_	2.9	1.7
CaO	62.7	50.3
MgO	1.50	4.98
SO_3_	3.22	2.41
Na_2_O	0.19	0.37
K_2_O	0.96	0.70
eqNa_2_O	0.82	0.83
Cl^−^	0.060	0.016
Portland clinker content GGBFS Gypsum	96 0 4	45 53 2

**Table 2:** Physical characteristics of CEMI and CEMIII cements.

Parameter	CEMI 42.5 R	CEMIII/A 42.5 N
Specific area (Blaine method), m^2^/kg True density, g/cm^3^	340 3.09	465 2.97
Setting time, minutes -initial -final	199 270	221 266

**Table 3:** Mechanical characteristics of CEM I and CEM III cements.

Parameters	CEMI 42.5 R	CEMIII/A 42.5 N
Compressive strength, MPa -after 2 days -after 28 days	29.3 55.1	13.7 50.7

**Table 4:** Mix composition of the test concretes.

	Concrete	Unit	B CEMI	B CEMIII	RB CEMI	RB CEMIII
Component						
CEM I 42.5 R		kg/m^3^	482		482	
CEM III/A 42.5 N		kg/m^3^		482		482
Water		dm^3^/m^3^	145			
w/c ratio		–	0.30			
Riverbed quartz sand 0–2 mm RB, riverbed 2–8 mm RB, riverbed 8–16 mm B, basalt 2–8 mm B, basalt 8–16 mm		kg/m^3^	662 – – 709 648	662 – – 709 648	663 610 558 – –	663 610 558 – –
Plasticizer BASF BV 18 Superplasticizer BASF Glenium SKY 591		% mc	0.90 2.20	0.90 2.35	0.90 2.20	0.90 2.35
Cement paste content Mortar content Coarse aggregate content		dm^3^/m^3^	300 550 450			
Slump (consistency)		mm	120–150			
Air content in concrete mix		% vol.	1.7–2.0			

**Table 5:** Initial properties and parameters of the test concretes.

Property	Unit	B CEMI	B CEMIII	RB CEMI	RB CEMIII
		B Basalt Coarse Aggregate		RB Riverbed Coarse Aggregate	
Bulk density ρ_o20°C_	kg/m^3^	2558.8	2533.2	2300.7	2315.6
Compressive strength f_c20°C_	MPa	84.9	96,2	77.0	87.4
Splitting tensile strength f_t20°C_	MPa	6.2	6.9	6.0	5.6
Modulus of elasticity E_20°C_	GPa	44.4	48.9	30.6	29.7
Permeability k_20°C_	m^2^	0.70 × 10^−17^	0.52 × 10^−17^	1.20 × 10^−17^	1.00 × 10^−17^
